# Ancient evolutionary origins of epigenetic regulation associated with posttraumatic stress disorder

**DOI:** 10.3389/fnhum.2014.00284

**Published:** 2014-05-13

**Authors:** Levent Sipahi, Monica Uddin, Zhou-Cheng Hou, Allison E. Aiello, Karestan C. Koenen, Sandro Galea, Derek E. Wildman

**Affiliations:** ^1^Center for Molecular Medicine and Genetics, Wayne State University School of MedicineDetroit, MI, USA; ^2^Department of Psychiatry and Behavioral Neurosciences, Wayne State University School of MedicineDetroit, MI, USA; ^3^National Engineering Laboratory for Animal Breeding and MOA Key Laboratory of Animal Genetics and Breeding, China Agricultural UniversityBeijing, China; ^4^Department of Epidemiology, University of North Carolina Gillings School of Global Public HealthChapel Hill, NC, USA; ^5^Department of Epidemiology, Mailman School of Public Health, Columbia UniversityNew York, NY, USA; ^6^Department of Obstetrics and Gynecology, Wayne State University School of MedicineDetroit, MI, USA

**Keywords:** posttraumatic stress disorder, epigenetics, molecular evolution, DNA methylation, mental disorders

## Abstract

Epigenetic marks, including DNA methylation, are modifiable molecular factors that may underlie mental disorders, especially responses to trauma, including the development of and resilience to posttraumatic stress disorder (PTSD). Previous work has identified differential DNA methylation at CpG dinucleotide sites genomewide between trauma exposed individuals with and without PTSD, suggesting a role for epigenetic potential—the capacity to epigenetically regulate behavior and physiology in response to lived experiences. The human species is characterized by an increased period of adaptive plasticity during brain development. The evolutionary history of epigenetic potential in relation to adaptive plasticity is currently unknown. Using phylogenetic methods and functional annotation analyses, we trace the evolution of over 7000 CpG dinucleotides, including 203 associated with PTSD, during the descent of humans in during mammalian evolution and characterize the biological significance of this evolution. We demonstrate that few (7%) PTSD-associated CpG sites are unique to humans, while the vast majority of sites have deep evolutionary origins: 73 and 93% were unambiguously present in the last common ancestor of humans/orangutans and humans/chimpanzees, respectively. Genes proximal to evolved PTSD-associated CpG sites revealed significant enrichment for immune function during recent human evolution and regulation of gene expression during more ancient periods of human evolution. Additionally, 765 putative transcription factor binding motifs (TFBMs) were identified that overlap with PTSD-associated CpG sites. Elucidation of the evolutionary history of PTSD-associated CpG sites may provide insights into the function and origin of epigenetic potential in trauma responses, generally, and PTSD, specifically. The human capacity to respond to trauma with stable physiologic and behavioral changes may be due to epigenetic potentials that are shared among many mammalian species.

## Introduction

The human species is characterized by an increased period of adaptive plasticity during brain development (Chugani, 1998; Sterner et al., 2012). This phenotypic plasticity enables individuals to respond in unique ways to environmental stimuli. Among other mechanisms, DNA methylation regulates phenotypic plasticity through epigenetic marks that are stable yet capable of experience-mediated dynamic change (Moore et al., 2013). Although DNA methylation may underlie mechanisms of phenotypic plasticity, the functional evolution of DNA methylation-mediated plasticity requires the presence of a genetic nucleotide substrate—the CpG dinucleotide. We thus refer to the presence of CpG dinucleotides required for phenotypic plasticity, “epigenetic potential.” The evolutionary history of this epigenetic potential is crucial to understanding various forms of phenotypic plasticity and related mental health outcomes.

Posttraumatic stress disorder (PTSD) and resiliency comprise two phenotypes that may arise subsequent to exposure to a traumatic event. Given that trauma is unpredictable yet probable within a lifetime, mechanisms of phenotypic plasticity in response to trauma are expected to exist, despite the prediction that such mechanisms will be costly (West-Eberhard, 2003). As such, we expect the epigenetic potential that underlies the capacity for trauma-induced phenotypic plasticity is possibly highly conserved. In humans, PTSD and trauma resiliency are associated with differential DNA methylation genomewide (Uddin et al., 2010; Smith et al., 2011) and, therefore, may be regulated by epigenetic potential.

Diagnostically, PTSD is a mental health disorder characterized by symptoms of intrusion, avoidance, and alterations in cognition and mood that cause distress or social impairment, last for more than one month, and are associated with a traumatic event consisting of direct or indirect exposure to actual or threatened death, injury, or sexual violation (American Psychiatric Association, 2013). Although PTSD diagnoses have historically been confined to humans, the capacity to express traumatic symptoms likely exists on other primate, and possibly non-primate mammalian, lineages. For example, abused chimpanzees have been reported to exhibit symptoms that in humans are considered characteristic of PTSD, including symptoms of avoidance, arousal, and dissociation (Bradshaw et al., 2008; Ferdowsian et al., 2011); elephants exposed to human violence have been documented exhibiting behaviors characterized by hyperaggression, abnormal startle response, depression, and asocial behaviors (Bradshaw et al., 2005); and rodents have been used as a model of PTSD, given their responses to trauma of social withdrawal and hyperarousal (Siegmund and Wotjak, 2006). These observations suggest that the capacity to express traumatic symptoms in response to extreme stress exists on non-human lineages within primate or mammalian clades and that this capacity may be an ancestral state (Horwitz and Wakefield, 2012).

Emerging evidence suggests that epigenetic variation may help explain observed differential susceptibility and resilience to PTSD in humans (Breslau et al., 1998). Indeed, recent studies (Uddin et al., 2010; Smith et al., 2011; Mehta et al., 2013), have demonstrated differential DNA methylation genomewide between individuals with PTSD and trauma exposed individuals without PTSD. Each of these studies demonstrated epigenetic dysregulation of immune system genes (Uddin et al., 2010; Smith et al., 2011)—a finding that is consistent with the knowledge that the hypothalamic-pituitary-adrenal axis modulates the immune system (Wong, 2002; Irwin and Cole, 2011) and findings from previous publications that differential gene expression patterns in genes involved in immune activation exist between PTSD-affected and -unaffected individuals (Segman et al., 2005; Zieker et al., 2007). PTSD, therefore, appears to be epigenetically regulated.

The evolutionary history of human epigenetic potential in relation to trauma-induced phenotypic plasticity would likely be informative of human phenotypic plasticity responses, generally, and PTSD, specifically. We hypothesized that the capacity to regulate behavioral, physiological, and psychological processes in response to traumatic experiences is mediated by epigenetic regulation at genetically inherited CpG loci that are largely conserved across mammalian species. Specifically, we expect that most of the CpG sites associated with the epigenetic regulation of PTSD will be largely conserved (that is, not unique to humans), but instead will have much more ancient origins. Here we test this hypothesis by tracing and characterizing the evolution of human CpG dinucleotides previously associated with DNA methylation patterns in whole blood that differentiate trauma exposed individuals with PTSD from those without PTSD. Doing so, we reveal the phylogenetic history of genetic CpG sites previously shown to be associated with the capacity to epigenetically regulate PTSD in humans and characterize different periods of the evolution of humans and other mammals.

## Materials and methods

### Emergence of CpG sites during human descent

Human CpG site annotation data was obtained from the Infinium HumanMethylation27 (HM27) DNA Analysis BeadChip by Illumina, which targets 27,578 CpG sites in more than 14,000 genes. Multiple sequence alignments were constructed using both Ensembl (v54, PECAN (Paten et al., 2008) for global multiple sequence alignments) and UCSC (Feb. 2009 assembly; Z-blast (Rosenbloom et al., 2010) for local multiple sequence alignments) using publicly available genomes of the following species: human (*Homo sapiens*), chimpanzee (*Pan troglodytes*), orangutan (*Pongo abelii*), macaque (*Macaca mulatta*), mouse (*Mus musculus*), rat (*Rattus norvegicus*), cow (*Bos taurus*), horse (*Equus caballus*), dog (*Canis lupus familiaris*), opossum (*Monodelphis domestica*), platypus (*Ornithorhynchus anatinus*), and chicken (*Gallus gallus*). The accelerated transformation maximum parsimony (ACCTRAN) algorithm (Farris, 1970), implemented in PAUP^*^4.0, was used to infer ancestral CpG site statuses for 19,711 alignable CpG sites. Only CpG sites for which alignments could be constructed using both UCSC and Ensembl and for which ancestral states were inferred unambiguously by parsimony (*CI* = 1) were utilized in downstream analyses. Using this process, the evolutionary history of 7202 CpG sites were unambiguously inferred along human descent within the context of a 12 species mammalian phylogenetic tree.

### A note on human descent branch labels

Throughout this paper we refer to branches on human descent in the following way, with divergence times estimates from Meredith et al. (2011) where applicable, and (Jameson et al., 2011) for primate nodes.

Human terminal: 7.2 million year period from the Last Common Ancestor (LCA) of humans and chimpanzees until present.
Human/chimpanzee stem: 10.8 million year period from the LCA of humans and orangutans (18.0 mya) to the LCA of humans and chimpanzees (7.2 mya).
Ape stem: 7.4 million year period from the LCA of humans and macaques (24.5 mya) to the LCA of humans and orangutans (18.0 mya).
Primate stem: 57.9 million year period from the LCA of humans and rodents (83.3 mya; here represented by rat and mouse) to the LCA of humans and macaques (25.4 mya).
Euarchontoglires stem: 8.7 million year period from the LCA of humans and Laurasiatherians (92.0 mya; here represented by dog, horse, and cow) to the LCA of humans and rodents (83.3 mya).
Placental stem: 98.0 million year period from the LCA of humans and opposums (190.0 mya) to the LCA of humans and Laurasiatherians (92.0 mya).
Theria stem: 27.8 million year period from the LCA of humans and platypus (217.8 mya) to the LCA of humans and opossum (190.0 mya).
Mammal stem: 106.7 million year period from the LCA of humans and chickens (324.5 mya) to the LCA of humans and platypus (217.8 mya).

### Evolution of PTSD-associated CpG sites during human descent

To gain insights into the evolutionary history and functional adaptive nature of PTSD, we inferred the evolutionary history of 203 PTSD-associated CpG sites identified by Uddin et al. (2010). Smith et al. (2011) and Mehta et al. (2013) have also published associations between PTSD and differential DNA methylation. Because samples utilized by these studies were chosen based on substantially different selection criteria—confined to PTSD in Uddin et al, whereas including selection for total life stress and childhood abuse in Smith et al. as well as Mehta et al.– we have chosen to analyze here only those PTSD-associated CpG sites identified by Uddin et al.

The following was completed on the 203 PTSD-associated CpG sites from Uddin et al. (2010) that mapped unambiguously onto our 12 species phylogenetic tree. Branch-specific rates of evolution during human descent were calculated using divergence dates estimates detailed above. Chi-square tests were utilized to analyze branch specific rates of evolution and are described in following:

1. A Chi-square test was used to test whether statistically significant differences exist between the branch distribution observed in the whole dataset (7202 sites) and the PTSD-associated subset (203 sites). For this test, expected and observed values represent, respectively, the distribution of changes of the whole data set and the PTSD-associated dataset.
2. A Chi-square test was used to test whether the number of PTSD-associated changes was branch specific (all branches considered). Respectively, expected and observed values represent an even distribution of changes and the actual distribution of changes in the dataset.
3. Given that there was a statistically significant difference according to branch, we used Chi-square tests to determine which branches differed from one-another (all individual branches compared to all other individual branches).

Putative transcription factor binding motifs (TFBMs) were identified that overlap with CpG sites of interest using the Genomatix MatInspector (Quandt et al., 1995; Cartharius et al., 2005) tool, with default settings. Input sequences included 60 base pairs up- and downstream of PTSD-associated CpG dinucleotide sites, as obtained from the Infinium HumanMethylation27 (HM27) DNA Analysis BeadChip by Illumina. The Database for Annotation, Visualization, and Integrated Discovery (DAVID) (Huang Da et al., 2009) was used with default settings to assess branch-specific enrichment of gene ontology terms and functional annotation clustering (FAC) among genes proximal to PTSD-associated CpG sites. Genes proximal to the 7202 total CpG sites for which evolution was unambiguously inferred were used as background for enrichment computations. Functional annotation clusters with Enrichment Scores greater than 1.3 were considered significant (Cartharius et al., 2005; Huang Da et al., 2009).

## Results

### Inference of PTSD-associated CpG dinucleotide phylogenetic history

**Figure 1:**
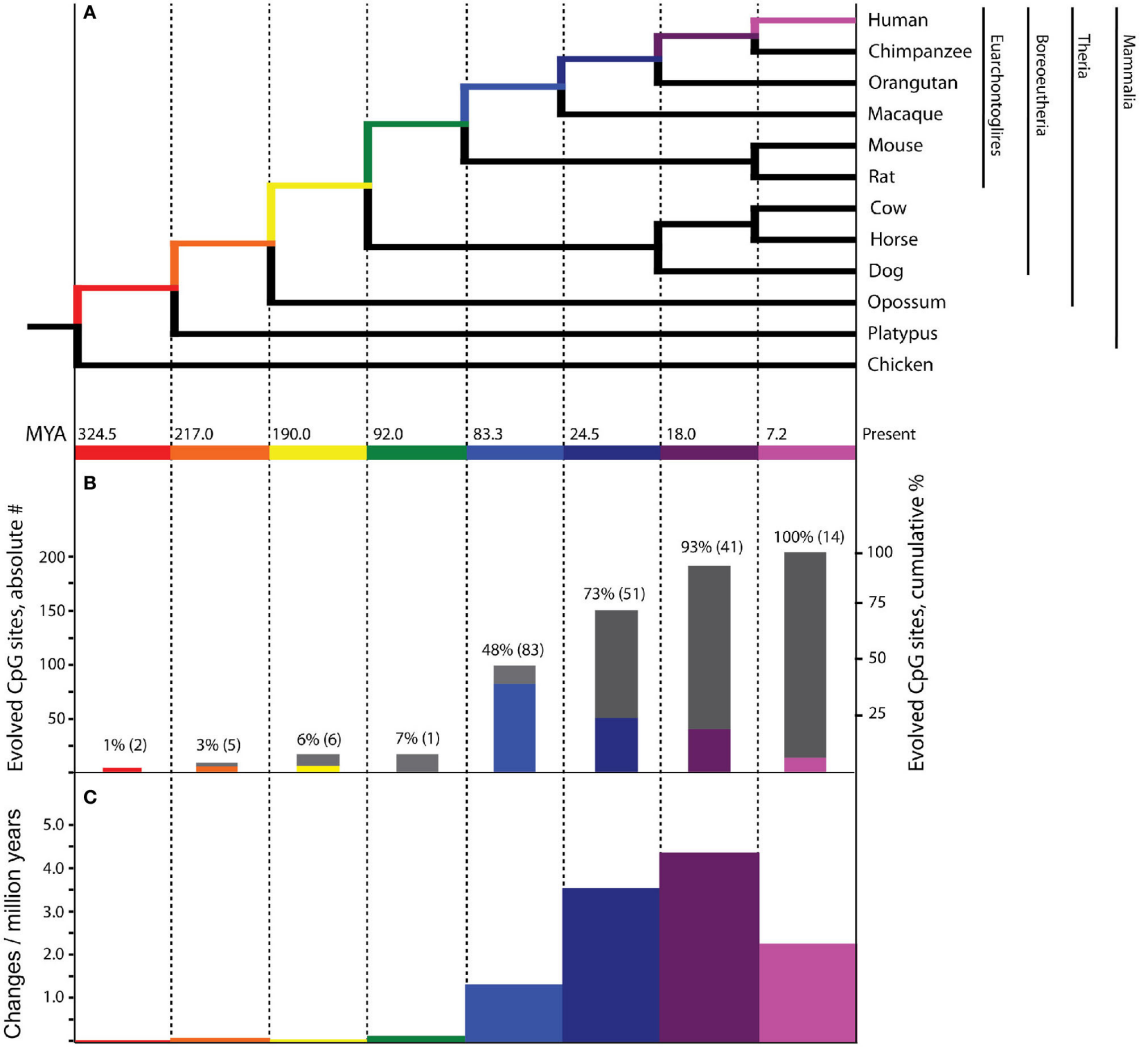
**Evolutionary history of PTSD-associated CpG dinucleotides.** Bars, tree branches, and time scales in panels **(A–C)** are color coordinated and vertically aligned. Red: mammal stem; Orange: Theria stem; Yellow: placental stem; Green: Euarchontoglires stem; Light Blue: primate stem; Dark Blue: ape stem; Purple: human/chimpanzee stem; Pink: human stem **(A)** Mammalian phylogenetic tree with species genomes utilized for the inference of molecular evolution here. Divergence dates at internal nodes along human descent, in millions of years ago. **(B)** The absolute number (colored bars) and cumulative percentage (gray bars) of PTSD-associated CpG sites evolved on branches of human descent, as inferred by parsimony. **(C)** Branch-specific rates of evolution of PTSD-associated CpG sites through human descent.

The phylogenetic histories of 7202 CpG dinucleotide sites from across the genome and assessed by the DNA methylation microarray were unambiguously mapped onto a mammalian phylogenetic tree (Figure 1A) by inferring ancestral states using a maximum parsimony method (Supplemental Table 1). Of these, 203 were previously identified to be differentially methylated in association with PTSD (Uddin et al., 2010).

Of the 7202 human CpG sites examined in extant mammals, 10.5, 52.0, 75.0, and 91.4% were present in the last common ancestor (LCA) of humans and rodents, Old World monkeys, orangutans, and chimpanzees, respectively. 8.6% of the CpG sites assessed evolved on the human terminal branch. The percentage of 203 PTSD-associated CpG sites evolved prior to the LCA of humans and rodents (7%), Old World monkeys (48%), orangutans (73%), and chimpanzees (93%) showed a branch distribution that did not differ statistically from that observed among the total 7202 assessed CpG sites (chi-square = 0.044, *df* = 7, *p* > 0.99) (Figure 1B). Among all CpG sites assessed as well as the subset of those associated with PTSD, there was a statistically significant association between branch of human descent and number of evolved CpG sites (Table 1; Total: chi-square = 7936.26, *df* = 7, *p* < 0.0001; PTSD subset: chi-square = 247.56 *df* = 7, *p* < 0.0001), such that the number of evolved PTSD-associated CpG sites was lowest on the mammal, Theria, placental, and Euarchontoglires stems, rose sharply beginning with the primate stem, peaked on the ape stem, and then decreased slightly on the human/chimpanzee stem and human terminal branch. The number of PTSD-associated CpG sites that evolved on the primate stem was significantly greater than any other branch of human descent assessed. (Table 1, Figure 1B).

**Table 1:**
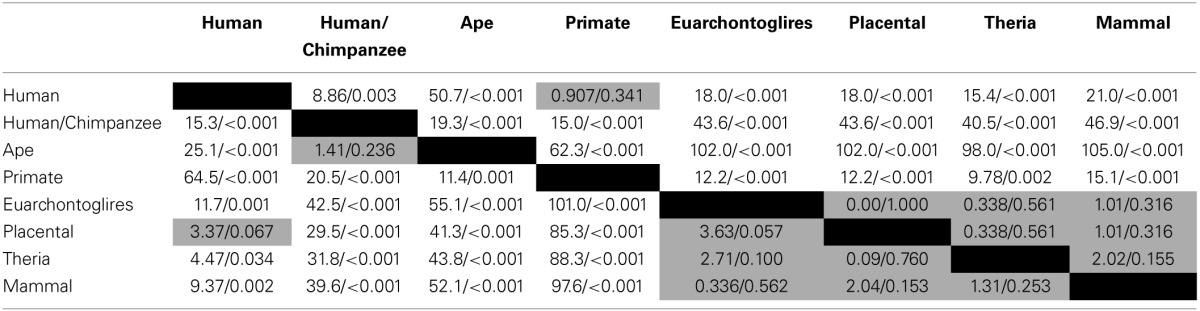
**Chi-square tests for branch-specific number and rate of evolved PTSD-associated CpG sites**.

Values are (Chi-square/p-value) and represent the result of a chi-square test with 1 degree of freedom. Cells above and below the diagonal represent evolutionary rate and raw number, respectively. Cells highlighted in gray represent tests that failed to reject the null hypothesis.

**Figure 2:**
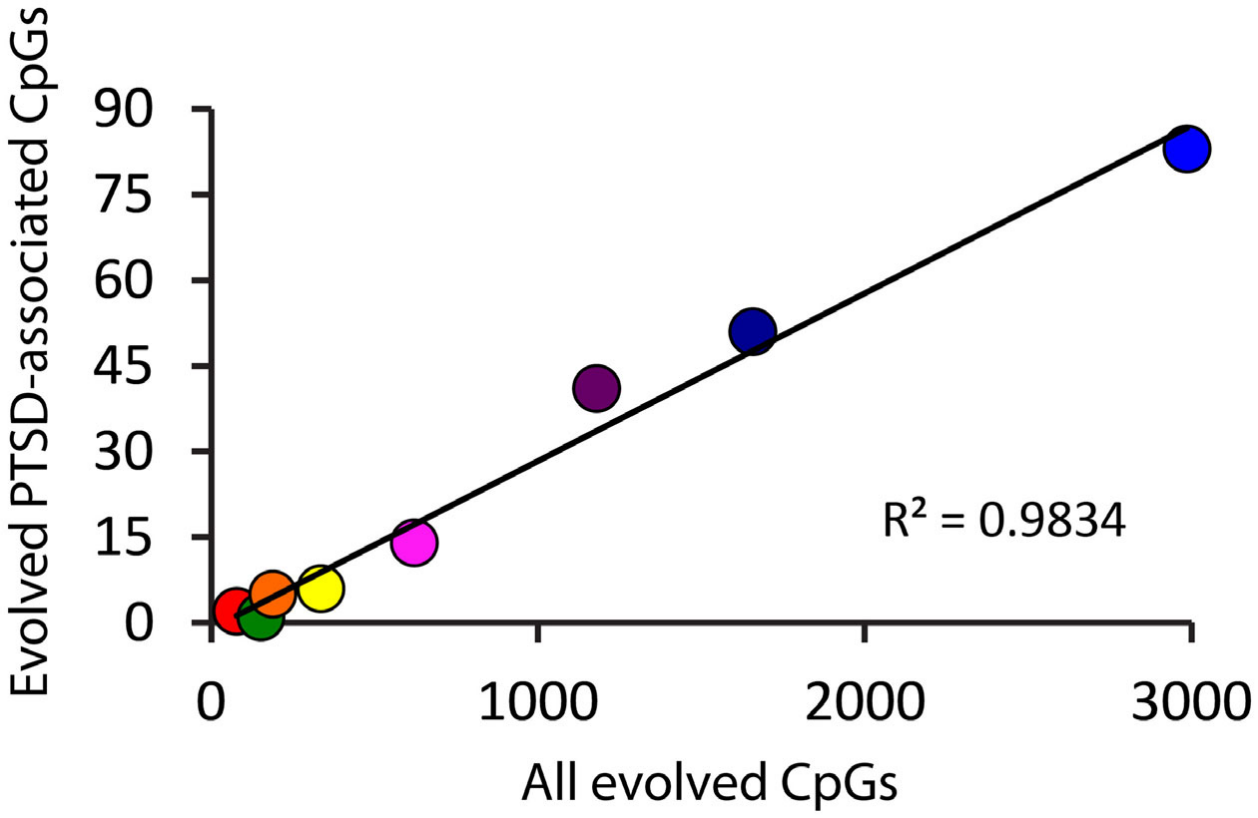
**Proportion of PTSD-associated CpG dinucleotides among all evolved CpGs scales linearly through human descent.** Color of circles corresponds to branches of human descent, as indicated in Figure 1. Red: Mammal stem; Orange: Theria stem; Yellow: placental stem; Green: Euarchontoglires stem; Light Blue: primate stem; Dark Blue: ape stem; Purple: human/chimpanzee stem; Pink: human stem.

Because there is high variability among the evolutionary time that spanned each of the branches assessed (Figure 1A), we computed branch-specific rates of evolution of total and PTSD-associated CpG sites (Figure 1C). The evolutionary rate of PTSD-associated CpG sites was statistically greater on the primate, ape, human/chimpanzee, and human terminal branches compared to that on the mammal, Theria, placental, and Euarchontoglires branches (Table 1, Figure 1C). PTSD-associated CpG sites evolved at the highest rate (6.9 changes/million years) on the ape stem (Table 1, *p* < 0.001 vs. all other branches). The rate of PTSD-associated CpG site evolution was lowest (Table 1) during human descent from the last common ancestor of all extant mammals 324.5 million years ago until the LCA of humans and rodents 92 million years ago (combined mammal + Theria + placental + Euarchontoglires stem lineages), during which time the evolutionary rate of PTSD-associated CpG sites never exceeded 0.2 changes/million years; the rate of PTSD-associated CpG evolution did not significantly vary between these branches of more ancient human descent (Table 1). Additionally, we observed a strong association between branch-specific evolution of all CpG sites and PTSD-associated CpG sites (Figure 2; *R*^2^ = 0.98338). That is, the proportion of PTSD-associated CpGs among all evolved CpGs was found to scale linearly through human descent.

### Gene functional enrichment analyses of PTSD-associated CpG dinucleotides

In order to shed light on selection pressures and biological significance of the evolution of PTSD-associated CpG sites, we tested for enrichment of gene ontology terms among genes located proximal (as predicted by beadchip annotation) to evolved PTSD-associated CpG sites. Background for this enrichment analysis was genes proximally located to the 7202 total CpG sites for which evolutionary history was unambiguously inferred (Supplemental Table 1). Enrichment analyses were completed for all 203 PTSD-associated CpG sites identified by Uddin et al. (2010) (Supplemental Tables 2–4) as well as on a branch-by-branch basis (Table 2). Annotation clusters with the five highest enrichment scores on each branch are listed in Table 2. The complete results of functional annotation analyses, including enriched GO terms (Supplemental Table 2) and significantly enriched Functional Annotation Clusters (Supplemental Table 3) can be found in Supplemental Tables; Significant FAC results (considered here to be those clusters with enrichment scores greater than 1.3 Huang Da et al., 2009) can be found in Supplemental Table 4. We detail several noteworthy findings below.

**Table 2:** **Top 5 functional annotation clusters of genes proximal to PTSD-associated CpG sites evolved on different branches of human descent**.

**Stem branch**	**Annotation cluster**	**Cluster rank**	**Enrichment score**
Human	Plasma membrane	1, 2, 3, 4	3.30, 2.98, 1.47, 1.45
	Immune response	5	1.30
Human/ Chimpanzee	Pathways in cancer	1	3.20
	B cell receptor signaling pathway	2	3.17
	MAPK/GnRH/Fc epsilon RI signaling	3, 4	2.85, 2.38
	Intestinal immune network (IgA)	5	2.09
Ape	Zinc finger/ion binding	1	3.75
	Plasma membrane	2	3.51
	Transcription/Zinc finger regions	3	3.29
	B cell/Fc epsilon RI/VEGF/GnRH/Natural killer cell signaling	4	2.68
	Long-term potentiation	5	2.46
Primate	Zinc finger region:C2H2	1, 2	7.74, 4.09
	Transcription regulation	3	4.01
	Plasma membrane	4	3.52
	Intracellular organelle lumen	5	3.24
Euarchontoglires	Src homology-3 domain	1	1.32
Placental	Transcription regulation	1, 2	2.88, 2.13
	Protein modification	3	1.84
	Cell fraction	4	1.35
	Regulation of apoptosis	5	1.31
Theria	Intracellular, non-membrane bound	1	2.80
	Transcription regulation	2	2.39
	DNA-binding/ helix-loop-helix	3	1.98
	Organelle lumen	4	1.94
	Sensory perception	5	1.42
Mammal	Negative regulation of transcription	1	1.71

In general, enriched annotation clusters involved transcriptional regulation throughout human descent, with the addition of immune-related annotations during more recent periods of human descent (Table 2). The human/chimpanzee stem lineage, during which the rate of PTSD-associated CpG evolution was greatest, was significantly enriched in annotation clusters involved in immune function, including adaptive (B cell receptor signaling pathway) and innate immune response (natural killer cell-mediated cytotoxicity) (Table 2). Likewise, the human terminal and ape stem lineages were enriched for immune response and B cell signaling annotation clusters, respectively (Table 2). Previous studies have found differential expression of immune function genes between trauma exposed persons with and without PTSD (Segman et al., 2005; Zieker et al., 2007). It is noteworthy, therefore, that the human terminal branch showed a FAC enriched for a number of immune functions including among others: immune response, immune response-activating cell surface receptor signaling pathway, T cell activation, lymphocyte activation, and leukocyte activation (Table 2).

In contrast, the primate stem lineage, on which the greatest number of PTSD-associated CpG sites evolved, had a FAC heavily associated with transcriptional regulation, including genes involved in epigenetic regulation: *HDAC1* and *HDAC11* (Table 2). Additionally, the annotation clusters with the three highest enrichment scores were found on the primate stem; each involved in transcriptional regulation and heavily enriched for zinc finger transcription factors (Table 2). It is noteworthy also that, with the exception of the human terminal, human/chimpanzee branch, and Euarchontoglires branch, each branch contained among its five most enriched clusters at least one involved in transcriptional regulation (Table 2).

### Transcription factor binding motif identification and characterization

As DNA methylation can regulate gene expression by structurally blocking transcription factor binding within gene promoter regions (Curradi et al., 2002), we sought to identify putative transcription factor binding sites that overlap with the 203 PTSD-associated CpG sites. Using a motif prediction algorithm, 765 total transcription factor binding motifs (TFBMs) were identified that overlap 196 PTSD-associated CpG sites. Of these, 765, 407, 188, and 22 putative TFBMs were identified that overlap with 196, 163, 107, and 21 unique PTSD-associated CpG sites at detection stringencies (mat_sim) of >0.80, >0.90, >0.95, and = 1, respectively (Supplemental Table 5). A stringency score of 1 indicates that the candidate sequence corresponds to the most conserved nucleotide at each position of the reference matrix. The 22 TFBMs with the highest stringency score (i.e., 1) spanned 21 different PTSD-associated CpG sites (Table 3). Two TFBMs with a stringency score of 1 spanned the CpG site cg13316424 of the gene *CIZ1*. Further, these 22 TFBMs were distributed among 9 different matrix families (Table 3). Notably, 4 of these TFBMs represent binding sites for transcription factor II B, which makes up the RNA polymerase II pre-initiation complex, while 2 are putative bindings sites of the transcription factor Beta2/NeuroD, which is responsible for neuron- and endocrine cell-specific gene expression. PTSD-associated CpG sites that overlap the RNA polymerase II pre-initiation complex binding motifs evolved on the ape (cg10498097/*MGC50811)*, primate (cg04033774/*GPSM2* and cg20318748*/NANP*), and placental (cg24673765/*HSPB6*) stem lineages, while those that overlap with Beta2/NeuroD binding motifs evolved on the ape (cg04587829/*FN3K*) and primate (cg00427635/*TBC1D21*) stem lineages. The branch-specific breakdown of the evolution of the 22 high stringency TFBMs is as follows: human (2), human/chimpanzee (5), ape (6), primate (5), placental (2), and therian (2).

**Table 3:** **Matrix families of TFBMs (mat_sim = 1) that overlap PTSD-associated CpG dinucleotides**.

**Matrix family**	**CpG**	**Gene**	**Stem branch**
C2H2 zinc finger transcription factors 2	cg27318281	*C18ORF37*	Human
	cg19047670	*CCND1*	Human/chimpanzee
	cg00962459	*PROKR1*	Ape
	cg03570766	*CATSPER1*	Ape
	cg12439773	*SLC22A6*	Theria
RNA polymerase II transcription factor II B	cg10498097	*MGC50811*	Ape
	cg04033774	*GPSM2*	Primate
	cg20318748	*NANP*	Primate
	cg24673765	*HSPB6*	Placental
Pleomorphic adenoma gene	cg06445611	*GABRR2*	Human/chimpanzee
	cg24505375	*AMAC1L2*	Human/chimpanzee
	cg13316424	*CIZ1*	Primate
	cg21835643	*RBPSUHL*	Theria
NeuroD, beta2, HLH domain	cg04587829	*FN3K*	Ape
	cg00427635	*TBC1D21*	Primate
TALE homeodomain class recognizingTG motifs	cg19531130	*ANGPTL5*	Human/chimpanzee
	cg01813965	*C16orf50*	Ape
Cart-1 (cartilage homeoprotein 1)	cg13316424	*CIZ1*	Primate
Human and murine ETS1 factors	cg06084117	*PLXNA4B*	Human/chimpanzee
	cg20792833	*PTPRCAP*	Placental
Vertebrate SNAD family of transcription factors	cg13471990	*ENTPD1*	Ape
Par/bZIP family	cg25293251	*GOLGA5*	Human

## Discussion

Epigenetic modifications provide a mechanism by which lived experiences can reprogram gene expression patterns and affect biological processes broadly. The epigenetic potential to respond to stress and trauma is likely conserved across mammalian species. As such, we predicted that the genetic elements (CpG dinucleotide sites) required for this epigenetic potential would be conserved. We have previously demonstrated that differential DNA methylation of CpG dinucleotides genomewide distinguish those with PTSD from trauma exposed individuals without PTSD (Uddin et al., 2010). Here, by tracing and characterizing the evolutionary history of 203 of these PTSD-associated CpG dinucleotide sites using functional annotation clustering and transcription factor binding motif identification, we provide evidence that the genetic substrate associated with divergent epigenetic responses to trauma is largely conserved across mammalian species. Among those PTSD-associated CpG sites for which we could unambiguously infer ancestral states, we demonstrate that (1) the majority evolved prior to the LCA of humans and Old World monkeys, (2) there is an enrichment among genes found proximal to evolved PTSD-associated CpG dinucleotides on all branches of human descent for annotation clusters involved in transcriptional regulation, while more recent branches of human descent (ape and human/chimpanzee stem branches, and human terminal branch) are enriched also for immune function-related annotation clusters; and (3) there is overlap with more than 800 putative TFBMs, the 22 most stringently selected of which fall into 9 TFBM families and evolved overwhelmingly on primate, ape, human/chimpanzee, and human stem terminal. Taken together, our data demonstrate that the human potential to epigenetically regulate traumatic responses may be shared with non-human primates and that the evolution of this capacity likely involved the targeting of TFBMs of many genes, including those involved in immune response and transcriptional regulation.

Functional annotation clustering and enrichment analysis of gene ontology terms identified enrichment of FACs involved in immune functioning that emerged throughout human descent generally, with over representation primarily concentrated during the most recent 15 million years of human evolution. It is notable that immune function dysregulation is a common finding among individuals with PTSD (Uddin et al., 2010; Smith et al., 2011)and evidence suggests that the immune system may play an important role in PTSD phenomenology (Segman et al., 2005; Zieker et al., 2007). Although not surprising, it is important to note that functional annotation enrichment among the 203 PTSD-associated sites tested here do not markedly differ from that observed in the larger dataset of 624 differentially methylated sites from Uddin et al. (2010). Indeed, the immune system interfaces closely with the HPA axis, a key regulator of the stress response. Glucocorticoids from the HPA axis trigger changes in expression of cytokines and inflammatory genes in leukocytes, while cytokine receptors in the hypothalamus trigger the release of glucocorticoids from the HPA axis in response to immune activation (Irwin and Cole, 2011). This complex interplay between the HPA axis and the immune system supports the evidence of immune involvement presented here. The identification of enriched immune function FACs may suggest recent evolutionary innovations or selection pressures on the ability of the immune system to respond to environmental exposures, possibly including trauma. Moreover, there is now clear evidence that the immune response is associated with a variety of mood disorders, and that cytokine activation in peripheral blood as well as brain cells, particularly microglia, underlie this association (Jones and Thomsen, 2013).

DNA methylation is thought to regulate gene expression, in part, by blocking the binding of transcription factor to binding sites (Iguchi-Ariga and Schaffner, 1989). Indeed, while several studies have identified differential methylation between trauma exposed individuals affected and unaffected by PTSD (Uddin et al., 2010; Smith et al., 2011), it is unclear what functional connection explains this association. Here, we identified approximately 800 putative transcription factor binding motifs that overlap PTSD-associated CpG sites, 22 of which met the highest detection stringency. Interestingly, 13 of these 22 putative binding sites overlapped with PTSD-associated CpG sites that evolved during relatively recent human evolution (human terminal = 2, human/chimpanzee stem = 5, ape stem = 6).

Our approach is novel in that it combines empirical insights of epigenetic variation to inform a genetic comparative analysis for the purpose of understanding human evolution. Classically, comparative molecular studies have compared genetic sequence or expression variation across species. However, recent work has compared DNAm variation across species (Zeng et al., 2012; Hernando-Herraez et al., 2013), with the hypothesis that species differences are due in part to gene regulatory differences created by species-specific promoter methylation. In contrast, we explore here a trait thought to be undergirded by epigenetic malleability and conserved across species.

A speculative interpretation of this data is that, in light of the strong conservation of PTSD-associated CpG sites, the potential to epigenetically regulate responses to extreme traumatic stress may well be adaptive. Depending on environmental circumstances, PTSD, or anxiety states more generally, can increase or decrease evolutionary fitness. Increased states of anxiety, although perhaps not ideal in many situations, can theoretically increase evolutionary fitness (Nesse, 2001, 2005). For example, genetic variants of catechol-O-methyltransferase (*COMT*) have been associated with a tradeoff between cognitive ability and behavioral measures of anxiety and stress resilience, giving rise to the so-called Worrier/Warrior selectionist model (Stein et al., 2002; Goldman et al., 2005; Mier et al., 2010). An epigenetically regulated developmental program that facilitates the plasticity required to assume an appropriate phenotype in response to environmental conditions may be similarly adaptive (Meaney, 2010). Given that there is strong conservation of PTSD-associated CpG sites among non-human primates, it may be the case that selective pressures have maintained the capacity to increase or decrease PTSD-like responses to trauma, including re-experiencing-, avoidance-, emotional numbing-, social withdrawal-, and hyperarousal-type symptoms in response to lived experiences and environmental exposures. We propose that the capacity to develop and epigenetically regulate a PTSD-like syndrome is potentially present in many mammalian species. Indeed, symptoms resembling PTSD and other mood disorders have been observed in chimpanzees (Bradshaw et al., 2008; Ferdowsian et al., 2011). We do not, however, expect that all species will respond to trauma exposure in the same way. Just as only a subset of trauma exposed humans develop PTSD symptoms, it is likely that individual variation within and among species is common in response to trauma exposure. Finally, the evolutionary history of the capacity to epigenetically regulate trauma responsivity is significant in light of the finding that environmentally induced epigenetic inheritance interacts with lifetime stress to alter brain development and genome activity (Crews et al., 2012).

Our study is limited by the number of CpG dinucleotides assessed by the methylation microarray and the number of publically available genomes utilized. While inclusion of additional genomes would have allowed the inference of phylogenetic histories with greater resolution, doing so would have limited the number of CpG sites analyzed in downstream analyses due to a larger number failing to meet our requirement of an unambiguous multiple sequence alignment. Inclusion of more species will provide valuable information and warrants further research. Indeed, because a large number of PTSD-associated CpG sites are shared among primates, the replication of this study with a more detailed primate phylogeny would be insightful. Additionally, characterization of the evolutionary history of the human capacity to epigenetically regulate trauma responses is limited by the incompleteness of PTSD-associated CpG sites assessed. Specifically, only 203 of 624 PTSD-associated CpG sites from Uddin et al. (2010) were both alignable and perfectly parsimonious, and therefore characterized here. That a majority of PTSD-associated CpG sites were unalignable or perfectly parsimonious is evidence of the dynamic nature of CpG evolution. Future studies are also needed to address the results here with regard to rapid deamination of methylated cytosines. Because of the high deamination mutation rate of methylated cytosines to thymines, there is an underrepresentation of CpG dinucleotides in the human genome. This may largely explain the observation that the vast majority of CpG evolution, including that of PTSD-associated CpGs, occurred more recently during human descent. Given this, it may be reasonable to examine the functional significance of those PTSD-associated CpG sites that evolved more anciently, assuming that they have been conserved because of a strong selection pressure to maintain them. The observation that immune system genes are enriched among the analyzed genes on more recent branches of human evolution does not necessarily imply that immune genes are not important in the evolution of non-human lineages. Finally, it should be noted that the identification of the PTSD-associated CpG sites analyzed here was performed using DNA methylation obtained from peripheral blood samples. While brain-derived DNA methylation data would be ideal to identify PTSD-associated biological markers, this is not feasible given a desire to use samples provided by living humans. However, individuals with PTSD have been shown to have reduced hippocampal area and hyperactivated amygdala relative to trauma exposed controls (reviewed in Pitman et al., 2012). Moreover, recent work suggests that there can be concordance between DNA methylation patterns in the blood and brain for stress-relevant genes (Klengel et al., 2013).

Interestingly, while we have traced and characterized the evolutionary history of PTSD-associated CpG sites, we also now have data on the evolution of non-PTSD-associated CpG sites. Comparing the branch-specific rates of evolution demonstrates that the rate of evolution of PTSD-associated CpG sites seems to scale linearly with human descent—suggesting that the evolutionary pattern observed, at least in terms of evolutionary rate, is a function of CpG evolution generally.

The vast majority of people experience a potentially traumatic event at some point in their lifetime, yet relatively few subsequently develop the diagnosable mental disorder PTSD. Elucidating the molecular and evolutionary underpinnings of severe trauma responses, such as PTSD, are required for the prevention and treatment of the disorder and for identifying factors involved in discrepancies in risk and resilience. Here, we contribute to the ongoing study of epigenetic influences on PTSD etiology and differential risk by having traced and characterized the evolution of genomic sites associated with the development of PTSD. Our data suggest that PTSD-associated CpG sites are found at highly predicted transcription factor binding sites, that the majority of such sites are shared by all primates and that the overrepresentation of PTSD-associated CpGs proximal to immune system-related genes may have disproportionately evolved during our more recent evolutionary history. Taken together, this data supports the hypothesis that the DNA sequences necessary for the epigenetic potential to develop a range of phenotypes in response to trauma (e.g., PTSD or resiliency) in humans have deep evolutionary origins and are widely conserved among mammalian species. It will be exciting to discover whether these epigenetic signals contributed to the evolution of human brain function and/or dysfunction.

## Author contributions

Levent Sipahi analyzed data and wrote the manuscript. Monica Uddin collected data, analyzed data, coordinated the study, and wrote the paper. Zhou-Cheng Hou analyzed data. Allison E. Aiello, Sandro Galea, and Karestan C. Koenen participated in the coordination of the study, collected data, and wrote the manuscript. Derek E. Wildman conceived the study, participated in the coordination of the study, collected data, and wrote the manuscript.

### Conflict of interest statement

The authors declare that the research was conducted in the absence of any commercial or financial relationships that could be construed as a potential conflict of interest.

## Supplementary material

Supplemental Table 1 **All evolved CpG dinucleotides (7202)**.

Supplemental Table 2 **Gene enrichment: gene ontology (all)**.

Supplemental Table 3 **Gene enrichment: functional annotation clustering (all)**.

Supplemental Table 4 **Gene enrichment: functional annotation clustering (Significant)**.

Supplemental Table 5 **Putative TFBMs at 4 stringencies**.
